# Pic Protein From Enteroaggregative *E. coli* Induces Different Mechanisms for Its Dual Activity as a Mucus Secretagogue and a Mucinase

**DOI:** 10.3389/fimmu.2020.564953

**Published:** 2020-11-17

**Authors:** Fernando Flores-Sanchez, Lucia Chavez-Dueñas, Javier Sanchez-Villamil, Fernando Navarro-Garcia

**Affiliations:** Department of Cell Biology, Centro de Investigación y de Estudios Avanzados del IPN (CINVESTAV-IPN), México DF, México

**Keywords:** autotransporter protein, MUC2, MUC5AC, mucus hypersecretion, mucin cleavage, intracellular calcium, Phospholipase C (PLC) pathway, mucus layer penetration

## Abstract

A hallmark of enteroaggregative *Escherichia coli* (EAEC) infection is the formation of an intestinal biofilm, which comprises a mucus layer with immersed bacteria. Pic is an autotransporter secreted by EAEC, and other *E. coli* pathotypes, and has been involved in two apparently contradictory phenotypes, as a mucus secretagogue and as a mucinase. Here, we investigated this Pic dual activity, mucus secretagogue capability and mucinolytic activity, in human goblet cells that secrete MUC2 and MUC5AC. Pic induced mucus hypersecretion directly in the goblet cells, without other intestinal cell types involved. At the same time, Pic exhibited strong proteolytic activity on the secreted mucins. These activities were independent since a mutation in the serine protease motif (PicS258I) abolished mucin degradation while maintaining the mucus secretagogue activity intact. Furthermore, deoxycholic acid (DCA)-induced mucins were proteolytically degraded when goblet cells were co-incubated with DCA/Pic, while co-incubation with DCA/PicS258I induced a synergistic effect on mucus hypersecretion. Pic was more efficient degrading MUC5AC than MUC2, but no degradation was detected with Pic inactivated at the active site by mutation or pharmacological inhibition. Remarkably, Pic cleaved MUC2 and MUC5AC in the C-terminal domain, leaving N-terminal subproducts, impacting the feature of gel-forming mucins and allowing mucus layer penetration by EAEC. Astonishingly, Pic stimulated rapid mucin secretion in goblet-like cells by activating the intracellular calcium pathway resulting from the PLC signal transduction pathway, leading to the production of DAG and releasing IP_3_, a second messenger of calcium signaling. Therefore, the dual activity of Pic, as a mucus secretagogue and a mucinase, is relevant in the context of carbon source generation and mucus layer penetration, allowing EAEC to live within the layer of mucus but also access epithelial cells.

## Introduction

Epithelial cells are covered by a thick layer of mucus, which serves as the first line of innate host defense. This thick layer consists of the gel-forming secreted mucins (mainly MUC2 and MUC5AC) synthesized by goblet cells (1). MUC2 is expressed in both mucus layers of colon, the outer unattached or the inner adherent. MUC5AC, is mainly present at the inner mucus layer of gastric mucosa, but is also expressed at some extent in the small intestine and colon (2, 3). Human MUC2, a major component of intestinal mucin, has a protein core of ~ 5,179 amino acids. Mucins contain a polypeptide backbone that is largely composed of serine, alanine, proline, glycine, and threonine (PTS domains), which are centrally located. N- and C-termini flanking the PTS domains are regions that contain an amino acid composition with virtually 10% cysteines (4). The N-terminus contains three von Willebrand D domains (D1 to D3) and one incomplete (D′), whereas the MUC2 C-terminus contains only one of such domain (D4), resembling the von Willebrand factor (5). During mucins secretion, MUC2 forms dimers in the endoplasmic reticulum by disulfide bonds between the C-termini by the cysteine-knot domain. The dimer is translocated into the Golgi apparatus, where PTS domains become densely O-glycosylated (80% of the MUC2 mass); this long and extended rod appearance (central protein core) resembles a bottle brush, with the brush bristles (O-glycans) extending in all directions (6). In secretory vesicles, low pH and high calcium concentration trigger the formation of N-terminal trimers linked by disulfide bonds (7). When the N-and C- termini intermolecular disulfide bonds have formed, a net-like structure is generated, forming longer branched structures (mucin multimers), which is protective physical barrier that prevents microorganisms and harmful substances from reaching the epithelial surface (8). Some colonic pathogens are skillful at breaking peptide bonds and glycosidic bonds in mucin structures conferring competitive advantages during infection. *V. cholerae* secretes a soluble dependent Zn-metalloproteinase called Hap that possesses mucinolytic activity and is required to pass through a mucin-containing gel (9)*. E. histolytica* secretes both an M60-like protease and a cysteine protease that degrade mucus; the latter disrupts the polymeric structure of colonic mucin and alter its protective function (10). StcE, a metalloprotease secreted by enterohemorrhagic *E. coli*, cleaves mucins, reducing the inner mucus layer and thereby promoted EHEC access and binding to the epithelium (11). Pathogenic *E. coli* also secretes SslE, a zinc-metalloproteinase that use its mucinase activity to cross the mucosal barrier and reach host cells (12). Understanding the molecular mechanisms that pathogens use to colonize the mucus layer is an important issue.

Among diarrheagenic *E. coli* strains, enteroaggregative *E. coli* (EAEC) is an excellent candidate to further understand how bacteria interact with mucin glycoproteins to colonize mucosal surfaces. A hallmark of enteroaggregative *Escherichia coli* (EAEC) infection is the formation of a biofilm, which comprises a mucus layer with immersed bacteria, on the intestines of patients (13). EAEC induces mild, but significant, mucosal damage on human intestinal specimens, including the mucosal surfaces of small and large intestinal (14, 15). Evidence suggests that mucus is a central component of the pathogenicity EAEC, as it avidly binds to intestinal mucin (16), volunteers fed EAEC strains excrete mucoid stools (17) and EAEC strains enhance mucus secretion from the mucosa (18). Hence, the formation of a mucoid biofilm may be related to the diarrheagenicity of EAEC and, perhaps, to its ability to cause persistent colonization and diarrhea.

EAEC secretes Pic (protein involved in colonization), which is a highly immunogenic autotransporter protein (19). As serine protease autotransporters of the *Enterobacteriaceae* (SPATE), Pic is a member of a group of exoproteins that contain a highly characteristic protease motif (GDSGSP) in the passenger domain (20). Despite their high levels of homology, SPATE proteins demonstrate distinct substrate specificities (21, 22). Unlike other autotransporters, Pic does not damage epithelial cells, it does not cleave fodrin or the host defense proteins embedded in the mucus layer (sIgA, lactoferrin, and lysozyme) (23). A functional analysis of the Pic proteolytic site showed its role in colonization by its mucinase activity, serum resistance, and hemagglutination. (24). The mucinolytic activity is dose-dependent and the Pic serine protease motif is required for this activity; since a site-directed mutagenesis in the motif of serine protease (PicS258I) did not show proteolytic activity (23). Interestingly, Pic binds mucin *via* the monosaccharide constituents of the oligosaccharide side chains of mucin, and mucin stripped of sialic acid decreases the Pic mucinolytic activity (23). Additionally, Pic targets a broad range of human leukocyte adhesion proteins and restrict its substrate specificity to O-linked glycan-rich glycoproteins (25).

Previously, we found that EAEC induced hypersecretion of mucus in an *in vivo* system, rat ileal loops. Remarkably, this mucus hypersecretion is abolished by using an isogenic *pic* mutant (EAECΔ*pic*) and induced with purified Pic protein (21). Other pathogens harboring the *pic* gene, such as *Shigella flexneri* and uropathogenic *E. coli* (UPEC), also caused mucus hypersecretion in ileal loops, and their isogenic *pic* mutants abrogate this activity (21). Therefore, Pic secretagogue activity is responsible for one of the pathophysiologic features of EAEC-mediated infection, the secretion of a mucus layer with immersed bacteria (21). Here we further investigate this dual activity of Pic, which apparently could encompass two contradictory effects: mucinolytic activity and mucus secretagogue. We used an *in vitro* culture model of goblet cells, to avoid paracrine effects by the other intestinal cell lines. The proteolytic and secretagogues activities of Pic were characterized using two relevant gel-forming mucins, MUC2 and MUC5AC. The use of antibodies against the C-terminal or N-terminal segments of MUC5AC and MUC2 allowed determining the probable cleavage site and the degraded segment of the mucin. Finally, the activation of second messengers and the use of specific inhibitors led to determination of the signaling pathways used by Pic to increase the rapid mucus secretion in goblet cells.

## Material and Methods

### Bacterial Strains

EAEC O44:H18 strain 042 was originally isolated from a child with diarrhea in Lima, Peru (17). The following EAEC variants were used: EAECΔ*pic*, HB101/p*Pic* (23), HB101/p*Pic*S258I (21), EAEC*pic*S258A (26), EAECΔ*pic*/p*Pic*, and EAECΔ*pic*/pTrcHis2B (in this study). All the strains were grown in LB medium at 37°C overnight and when required ampicillin (50 µg/ml), kanamycin (50 µg/ml), or tetracycline (15 µg/ml) were added.

### Cell Culture

LS174T cells (ATCC CL-188) were cultured in DMEM (Dulbecco modified Eagles minimal essential) medium supplemented with 10% fetal bovine serum (FBS) (Biowest, Nuaillé, France), 1% non-essential amino acids, penicillin (100 U/ml), and streptomycin (100 µg/ml) at 37°C in a humidified atmosphere at 5% CO_2_.

### Obtaining Pic and PicS258I

Pic and PicS258I proteins were partially purified as previously reported (21). Briefly, minimal clones HB101/p*Pic* and HB101/p*Pic*S258I were grown in LB medium supplemented with 15 µg/ml tetracycline for 16 h in agitation. Bacteria were centrifuged at 2240 ×*g* for 20 min and the supernatants were filtered through a 0.2 micrometers filters and concentrated 150 times using an Amicon filter with a cut-off of 100 kDa (Millipore, Bedford, MA), all the time kept in cold-ice.

### EAECΔ*pic* Complementation

The *pic* gene was amplified from genomic DNA from EAEC 042 using the following oligonucleotides: Fw 5’- CCG CTC GAG CAT GAA TAA AGT TTA TTC TCT TAA ATA TTG CC -3’ and Rv 5’- CCG GAA TTC TCA GAA CAT ATA CCG GAA ATT CGC GTT -3’. The PCR product of 4,110 bp was purified using the QIAquick Gel Extraction Kit (Qiagen, Hilden, Germany) and digested with *Xho*1 and *EcoR*1 enzymes, and cloned in the inducible vector pTrcHis2B (Thermo Fisher Scientific, Rockford, IL). For the complementation, EAECΔ*pic* bacteria were transformed with plasmid pTrcHis2B/*pic* to obtain the construction EAECΔ*pic*/p*Pic*. In posterior experiments, EAECΔ*pic*/p*Pic* was induced with 1 mM IPTG.

### Mucin Secretion

LS174T cells were cultivated in 48-well plates (80,000 cells per well) for 72 h, until confluency was reach. Cells were kept for 17 h in DMEM medium without SFB or antibiotics, washed exhaustively with fresh DMEM medium without supplements. Cells were incubated with 350 µl of DMEM medium containing the different treatments: Pic, PicS258I or deoxycholic acid (DCA) for 4 h at 37°C in 5% CO2. Finally, the supernatants were recovered and centrifuged at 110 ×*g* for 10 min.

### Mucin Detection by ELISA

MUC5AC secretion was detected by ELISA. Fifty µl of supernatants from LS174T cells diluted in 150 µl of binding buffer (0.125 M NaHCO_3_ and 0.125 M Na_2_CO_3_, pH 9.5) were coated on the ELISA plates (Corning, New York) for 16 h at 4°C. Plates were washed three times with 0.05% PBS-Tween and blocked with 3% BSA for 2 h at room temperature. After another round of washing, plates were incubated with 120 µl of a monoclonal anti-MUC5AC antibody (45M1, diluted 1:200; Thermo Fisher Scientific) for 5 h at 37°C. Then, plates were washed again and incubated for 1 h with a secondary antibody, mouse anti-IgG coupled to horseradish peroxidase (HRP) diluted 1:5,000 (Invitrogen, Eugene, OR). After washing, the immunoreactivity was developed by incubating the plates with 10 mg de OPD (*o*-Phenylenediamine dihydrochloride) (Sigma-Aldrich, St. Louis, MO) diluted in 25 ml of substrate buffer (0.05 M NaH_2_PO_4_, 0.025 M citric acid and 0.05% H_2_O_2_, pH 4). After 15 min, the enzymatic reaction was stopped with 30 µl of 2 N H_2_SO_4_. Optical density was read at 490 nm using an ELISA reader (iMark Microplate Reader, Biorad Labs, Hercules, CA).

### MUC2 and MUC5AC Degradation Assays

LS174T cells were growth in 90 mm plates (Corning) in DMEM medium supplemented with 10% FBS until confluency. After washing the cells with PBS at 37°C, they were removed with a cell scraper using PBS and protease inhibitors Complete 1× (Roche Diagnostics, Mannheim, Germany) and lysed through two freeze and thaw cycles. Lysates were centrifuged at 21,690 ×*g* at 4°C for 10 min, supernatants were recovered and dialyzed at 4°C for 4 h with cold PBS using a dialysis membrane (Spectra/Por^®^ Dialysis membrane) of 12-14 MWCO (Thermo Fisher Scientific).

For MUC2 and MUC5AC degradation assays, 20 µg of the soluble fraction of lysates were incubated with 2 µg of Pic, PicS258I or Pic preincubated with 1 mM of phenylmethylsulfonyl fluoride (PMSF) or 1 µg of Proteinase K (PK) at 37°C for 2 h and pH 7.2, in a reaction volume of 50 µl. Due to antibody nature, for MUC2 detection the samples were denatured with Laemmli buffer with β-mercaptoethanol, while for MUC5AC detection Laemmli buffer was used without β-mercaptoethanol. Mucins were separated by SDS-agarose gels electrophoresis as described previously (27). Briefly, a 1 cm plug of 12% polyacrylamide was cast using 10 x 8 cm glass plates. After polymerization, it was overlaid by a 1% agarose gel prepared in a running buffer (50 mM Tris, 384 mM glycine, and 0.1% SDS) with 30% glycerol. The samples were electrophoretic run at 13 mA for 1.5 h. The samples were transferred onto 0.45 µm PVDF membranes (Thermo Fisher Scientific, Rockford, IL) at 320 mA at 4°C for 2 h. Alternatively, samples were separated in SDS-PAGE, using gels consisting of 4% stacking gel and 5% separating gel, and transferred to PVDF membranes. PVDF membranes were blocked with 5% nonfat dried milk at room temperature for 2 h. For MUC2 detection, the PVDF membranes were incubated overnight with a monoclonal anti-MUC2 antibody Ccp58 (diluted 1:500, Thermo Fisher Scientific) or H-300 (diluted 1:1,000, Santa Cruz Biotechnology, Santa Cruz, CA), while for MUC5AC detection, two kind of antibodies were used and PVDF membranes were incubated overnight with either a monoclonal anti-MUC5AC 45M1 (1:500, Thermo Fisher Scientific) or a polyclonal anti-MUC5AC H-160 (1:1,000, sc-20118) (Santa Cruz Biotechnology). Then, PVDF membranes were washed five times with PBS-Tween and incubated for 1 h with mouse anti-IgG or rabbit anti-IgG (1:10,000, Invitrogen; 1:20,000 Novex, San Diego, CA), respectively. Finally, PVDF membranes were washed nine times and developed with Immobilon Western Chemiluminescent HRP Substrate (Millipore) using the Odyssey Fc imaging system (LI-COR Biosciences; Lincoln, NE).

### Obtaining Secreted Mucin MUC5AC

LS174T cells were growth in 90 mm plates until confluency and then kept in 12 ml of DMEM without FBS and antibiotics at 37°C for 17 h. After this time, supernatants were recovered and centrifuged at 110 ×*g* at 4°C for 10 min. Supernatants (15 ml) were concentrated 150 times using Amicon filters of 100,000 MWCO (Millipore). Mucins were quantified using Bradford assay and stored at -70°C until their use.

### Degradation Assays of Secreted MUC5AC

For degradation assays of secreted MUC5AC, 0.5 µg of mucin was incubated with Pic, PicS258I or PMSF-preincubated Pic, or 1 µg of Proteinase K at 37°C (pH 7.2) for 2 h in a final reaction volume of 50 µl. Then, samples were dropped on nitrocellulose membrane (0.4 µm) using a Dot-blot apparatus (Bio-Dot apparatus, Biorad, Richmond, CA). Samples were analyzed by immunoblot using the anti-MUC5AC 45M1 (1:500).

### Immunofluorescence Assays

LS174T cells (60,000) were growth in each well of an 8-wells Nunc Lab-Tek (Thermo Scientific) at 37°C for 48 h in a humidified atmosphere with 5% CO_2_. Washed cells, were kept for 2 h in DMEM medium without FBS and antibiotics. Then, washed cells were replaced with 300 µl of fresh DMEM medium without supplements and incubated with 5 µg/ml of Pic, PicS258I or PMSF-preincubated Pic, or 0.125 mM DCA at 37°C for 4 h with 5% CO_2_.

After incubation time, culture medium was removed and cells were fixed with 4% paraformaldehyde (PFA) for 20 min at room temperature. Cells were not permeabilized but blocked with 3% BSA for 30 min. Washed cells were incubated with 120 µl of the monoclonal anti-MUC5AC antibody 45M1 (1:100) at 37°C for 3 h. Cells were washed seven times and incubated 1 h with Biotin-SP (long spacer) AffiniPure Goat Anti-Mouse IgG (1:800, Jackson ImmunoResearch Labs, West Grove, PA) secondary antibody followed by dichlorotriazinylamino fluorescein (DTAF)-conjugated streptavidin (1:800, Jackson ImmunoResearch Labs). Finally, cells were stained with Rhodamine Phalloidin (1:80, Invitrogen) to stain F-actin and TO-PRO-3 (1:400, Invitrogen) to stain nuclei. Then, slides were washed and mounted using VECTASHIELD (Vector Laboratories, Burlingame, CA) antifade mounting medium and analyzed using a Leica confocal microscopy TCS SP8 (Leica Microsystems, Wetzlar, Germany).

### Levels of Intracellular Calcium in LS174T Cells

Intracellular calcium concentration was determined as previously reported (28). Briefly, 60,000 LS174T cells were cultured in each well of an 8-wells Nunc Lab-Tek (Thermo Fisher Scientific) at 37°C for 4 h in a humidified atmosphere with 5% CO_2_. Cells were kept in DMEM without supplements for 2 h and loaded with 2 µM Fluo-4AM (Invitrogen) in Ringer buffer (10 mM HEPES, 145 mM NaCl, 5 mM KCl, 1 mM MgCl_2_, 2 mM CaCl_2_, pH 7.3) at 37°C for 45 min. Then, the medium was removed and fresh DMEM medium without supplements was added and incubated for 30 min. After this incubation, cells were incubated for 4 h with 5 µg/ml of Pic, PicS258I or 0.125 mM DCA. Finally, cells were stained with DAPI (Sigma-Aldrich) and the preparations were analyzed by a Leica confocal microscopy TCS SP8 (Leica Microsystems).

### PKC Activation Assays

For PKC activation by Pic experiments, 500,000 LS174T cells were growth in 35 mm plates (Jet Biofil, Guangzhou, China) for 48 h. Before experiments, cells were kept in DMEM medium without supplements for 19 h. Washed cells were incubated with 5 µg/ml of Pic, PicS258I or 2 µM Phorbol 12-myristate 13-acetate (PMA) at different times. Then, cells were washed with PBS at 37°C and lysed with a NP40 lysis buffer (50 mM Tris pH 8, 150 mM NaCl, 1% NP40, 2 mM NaF, 5 mM Na_3_VO_4_) and protease inhibitors Complete 1×. Lysates were centrifuged at 14,000 rpm and then the soluble fraction was recovered. Twenty µg of total protein were denatured with Laemmli buffer containing β-mercaptoethanol and then separated by SDS-PAGE and analyzed by Western blot using either anti-PKCδ-total (sc-8402) or p-PKCδ (sc-374613) (Santa Cruz Biotechnology).

For PLC inhibition, LS174T cells were preincubated with 20 µM inhibitor U73122 (Sigma-Aldrich) for 45 min.

### Bacterial Penetration of the Mucin Layer

To determine if EAEC bacteria use Pic to cross the mucin barrier, these experiments were performed as previously reported (29), with some modification. Briefly, in the inserts of Transwells, 12 mm of dimeter with 3 µm pores (Corning), were layered 200 µl of secreted mucin from LS174T cells (0.025 µg/µl) and then mounted in 24-well plates, which contained 1,200 µl of LB medium. Previously, EAEC, EAECΔ*pic*, EAECΔpic/p*Pic*, EAECΔ*pic*/pTrcHis2B, or EAEC*pic*S258A strains were growth in LB medium with the appropriate antibiotics at 37°C for 16 h. Then, 20 × 10^6^ CFUs of each bacterial strain (10 µl) were inoculated in each mucin-containing well insert and incubated at 37°C for 2 h.

After the incubation time, the LB in the bottom chamber was recovered and double serial dilutions were performed. Each sample was plated in LB-agar plates and incubated at 37°C overnight, and CFUs were quantified.

### Statistical Analysis

Data are shown as mean ± SEM (standard error of the mean). Comparison among different groups were performed using one-way ANOVA with Dunnet’s *post hoc* test using the GraphPad 8 software (La Jolla, CA), a value of *p <*0.05 was considered as a significant statistical difference.

## Results

### Pic Directly Induces Mucus Hypersecretion in Human Goblet Cells

To evaluate the mucus hypersecretion by LS174T cells, we used a well-known secretagogue, deoxycholic acid (DCA). LS174T cells were incubated with different DCA concentrations for 4 h and then supernatants were collected. MUC5AC and MUC2 secretion were measured by ELISA using specific anti-MUC5AC (45M1) and anti-MUC2 (Ccp58) antibodies. Supernatants from untreated LS174T cells were used as a negative control and to determine basal MUC5AC and MUC2 secretion. DCA increased the secretion of both MUC5AC and MUC2 at all concentrations used (**Figure S1A** and **S1B**). However, DCA-induced MUC5AC secretion reached a plateau at all concentrations used: 139% at 0.062 mM, 148% at 0.125 mM and 131% at 0.25 mM of DCA, using the basal secretion as 100% of secretion (**Figure S1A**). Whereas, DCA-induced increase in MUC2 was concentration-dependent and reached higher levels that of MUC5AC secretion: 106% at 0.062 mM, 285% at 0.125 mM and 369% at 0.25 mM DCA (**Figure S1B**).

**Figure 1:**
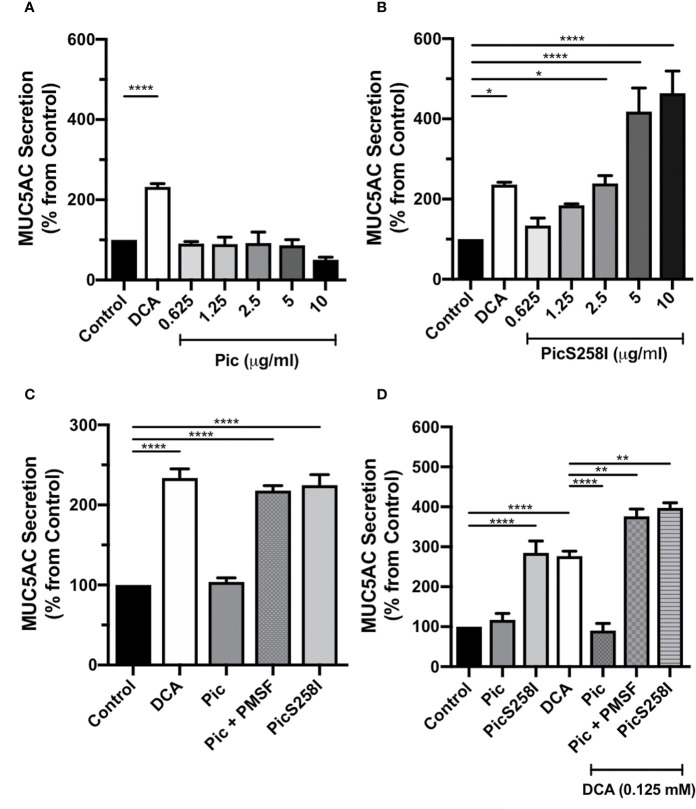
Pic induces MUC5AC rapid secretion directly on goblet cell-like, independent of its serine protease motif. **(A, B)** Pic degradation avoids detection of mucin hypersecretion. LS174T cells were stimulated with different concentrations of Pic **(A)** or PicS258I **(B)** at 37°C for 4 h. Deoxycholic acid (DCA, 0.125 mM) was used as a positive control, a known mucin secretagogue on LS174T cells. **(C)** Pic induces mucin hypersecretion independently on the serine protease motif. LS174T cells were stimulated with 2 µg/ml of Pic, PMSF-preincubated Pic or PicS258I or 0.125 mM DCA, at 37°C for 4 h. **(D)** Either Pic mutant or Pic with PMSF plus DCA increase synergistically MUC5AC secretion. LS174T cells were co-stimulated with 0.125 mM DCA and 2 µg/ml of Pic, PMSF-preincubated Pic or PicS258I, or stimulated with these proteins but without DCA, at 37°C for 4 h. After interactions, supernatants were recovered and MUC5AC was detected by ELISA using a monoclonal anti-MUC5AC antibody (45M1) or an anti-isotype antibody and developed with a secondary antibody coupled to HRP. Data are shown as mean ± SEM of at least 3 independent experiments. Statistical analysis was performed using one-way ANOVA with Dunnet’s *post hoc* test, *p < 0.05%, **p < 0.01%, ****p < 0.0001%.

To determine whether Pic is a mucus secretagogue that acts directly on goblet cells, only this cell type was cultured alone to avoid the epithelial cytokines produced by enterocytes. Thus, LS174T cells were incubated with Pic protein at different concentrations (0.75, 1.5, 2.5, 5, and 10 µg/ml) for 4 h, and Pic-induced MUC5AC secretion in cell supernatants was compared to that of the positive control DCA-induced secretion and using untreated conditions as a negative control. Unexpectedly, Pic was unable to increase MUC5AC secretion from these cells at any of the concentrations used, similar to that of DCA (0.125 mM) did (**Figure 1A**). The inability to detect Pic-induced MUC5AC secretion in goblet-like cell supernatants suggested that Pic did not act as a direct secretagogue but also raised the possibility that Pic could exhibit secretagogue properties concomitant with its protease activity, which could account for the low availability of intact mucin. To examine the second possibility, LS174T cells were incubated for 4 h with increasing concentrations of PicS258I. Surprisingly, ELISA analysis showed that PicS258I caused a strong increase in MUC5AC secretion as compared with the basal secretion from untreated cells (**Figure 1B**) or from cells treated with native Pic. At a concentration of 2.5 µg/ml, PicS258I significantly increased MUC5AC secretion, with levels reaching twice (220%) the amount observed under basal conditions. At higher concentrations (5 and 10 µg/ml), PicS258I induced increases in MUC5AC secretion that were four and five times (406% and 540%, respectively) that of untreated cells (**Figure 1B**). These data suggest that Pic acts as a potent mucus secretagogue with concomitant potent mucinase activity, resulting in mucin degradation.

### Pic Dual Activity as a Mucin Secretatogue and a Mucinase; the Latter can Hide the Activity of the Former

To determine the role of Pic serine protease activity in mucin hypersecretion, LS174T cells were incubated with native Pic that had been previously inactivated with PMSF, an irreversible serine protease inhibitor. MUC5AC secretion levels in the supernatant of these cells were compared with those originating from cells incubated with native Pic or PicS258I (2 µg/ml), using 0.125 mM DCA as a positive control and untreated cells as a control of basal secretion. PMSF-preincubated Pic (2 µg/ml) induced a two-fold increase in MUC5AC secretion (218%) as compared with basal secretion (100%). The MUC5AC secretion levels resulting from preincubation of Pic with the serine protease inhibitor equaled those achieved with the active site mutant PicS258I (224%). In contrast, native Pic incubation did not induce an increase in MUC5AC secretion levels, which were similar to those of untreated cells (**Figure 1C**). Since the proteolytic activity of the serine protease motif of Pic could interfere with LS174T cell viability, we evaluated cell viability using an MTT assay after treatment with DCA, Pic, and PicS258I. Untreated cells were used as a negative control, and Triton X-100-treated cells were used as a positive control of reduced cell viability. Except for cells treated with Triton X-100, all conditions exhibited high cell viability, suggesting that Pic protease activity does not damage goblet-like cells (**Figure S2**).

Since our data strongly suggest that the induction of mucin secretion and the degradation of mucin may represent two concomitant functions of Pic, we decided to clearly examine this dual role. LS174T cells were costimulated with DCA (0.125 mM) and either native Pic, PicS258I, or PMSF-preincubated Pic (2 µg/ml). Additionally, LS174T cells were incubated with native Pic or PicS258I alone, and DCA alone was used as a positive control. After 4 h of treatment, cell supernatants were recovered and analyzed for MUC5AC secretion by ELISA. Remarkably, costimulation with DCA and native Pic did not induce an increase in MUC5AC secretion, revealing similar levels as those measured in untreated cells. In contrast, DCA treatment alone induced an almost three-fold increase in MUC5AC secretion levels (277%) as compared to basal secretion conditions, while secretion levels that were similar to those of negative control were observed following native Pic treatment, which were comparable to the levels induced by DCA/Pic costimulation (**Figure 1D**). Interestingly, costimulation with DCA and PMSF-preincubated Pic induced an almost four-fold increase in MUC5AC secretion (377%), which was higher than the secretion induced by DCA alone (277%). Similarly, LS174T cells costimulated with DCA and PicS258I were exhibited a four-fold increase in MUC5AC secretion levels (398%), which were higher than those induced by PicS258I alone (284%) (**Figure 1D**), indicating a synergistic or semiadditive effect of DCA and Pic inactivation, either by pharmacological inhibition or mutations at the active site.

Taken together, these data indicate that Pic increases mucin secretion in a serine protease independent fashion and degrades such secreted mucins in a serine protease motif dependent manner.

### Pic Efficiently Cleaves Mucin at the C-Terminal Domain

**Figure 2:**
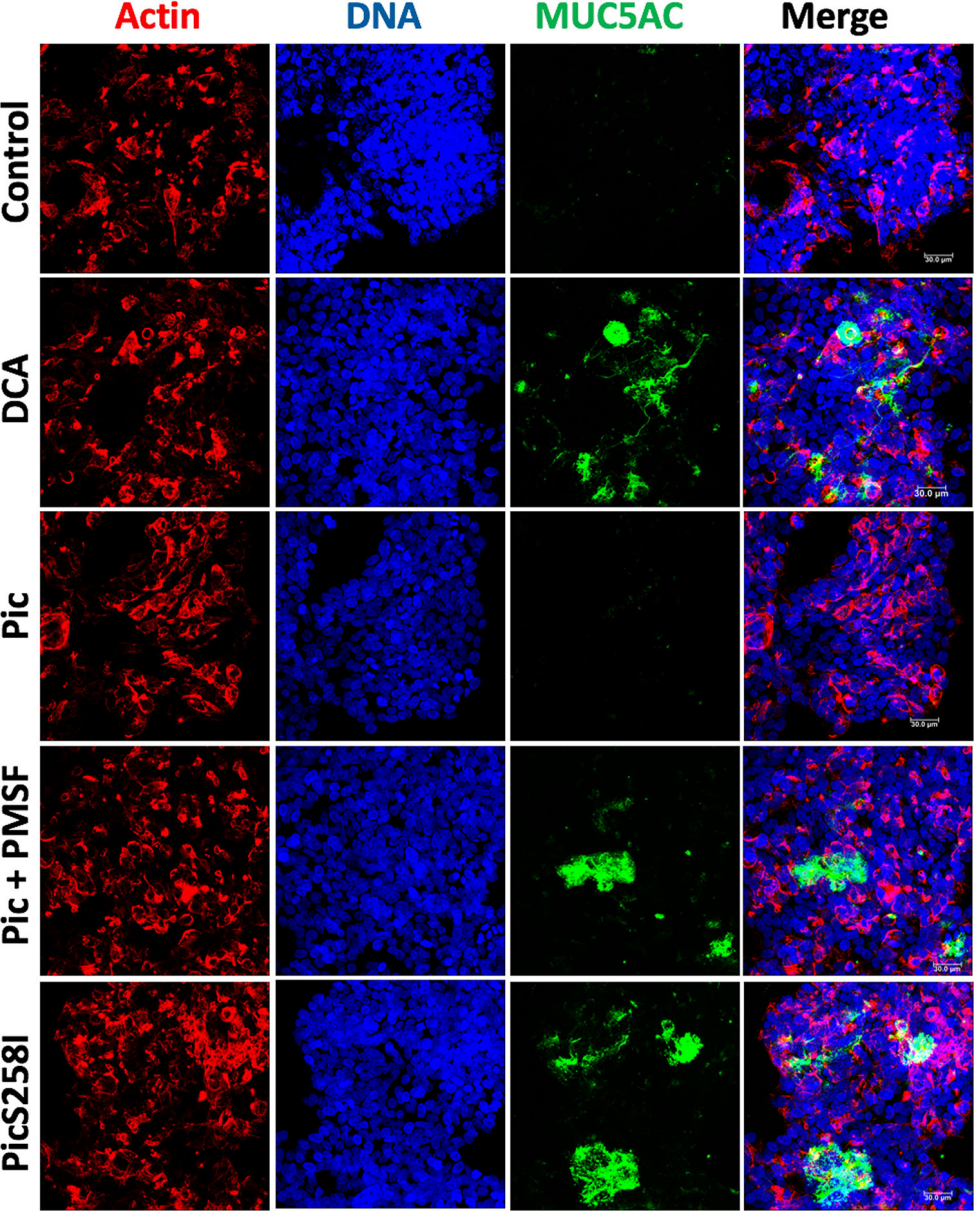
Pic dual activity detected by secretion and degradation of MUC5AC. LS174T cells were stimulated with 5 µg/ml of Pic, PMSF-preincubated Pic or PicS258I, at 37°C for 4 h. DCA (0.125 mM) was used as a positive control. After interactions, cells were fixed but not permeabilized and MUC5AC secretion was detected by immunofluorescence using a monoclonal anti-MUC5AC antibody (45M1) followed by a secondary antibody coupled to fluorescein-biotin (green). Actin was stained rhodamine phalloidin (red) and nuclear DNA with TO-PRO-3 (blue). Preparations were analyzed and recorded with a confocal microscopy, Leica TCS SP8 (Leica Microsystems).

To characterize the degradation of human mucins secreted by goblet-like cells, we used LS174T cells to measure MUC5AC and MUC2 degradation by Pic protein. First, we visualized Pic-induced MUC5AC secretion and degradation by confocal microscopy. LS174T cells were stimulated for 4 h with native Pic, PMSF-preincubated Pic or PicS258I. DCA was used as a positive control and untreated cells were used as a negative control. After stimulation, to visualize extracellular secreted mucins, LS174T cells were fixed in the absence of permeabilizing agents and then immunolabeling with anti-MUC5AC antibodies (45M1), followed by fluorescein-labeled (green) secondary antibodies. Cells were visualized by staining with rhodamine-phalloidin to detect F-actin and TO-PRO-3 to detect nuclear DNA. Small traces of MUC5AC were detected in the extracellular compartments of untreated cells, while in cells stimulated with the secretagogue DCA, it was possible to detect MUC5AC mucin secreted by the goblet-like cells. As expected, no MUC5AC was detected in LS174T cells treated with native Pic, since the secreted mucin was also degraded by Pic. Blocking the serine protease motif by the specific inhibitor PMSF or through site-directed mutagenesis inhibited mucin degradation, and therefore, the anti-MUC5AC antibody was able to detect MUC5AC secretion by goblet-like cells (**Figure 2**).

**Figure 3:**
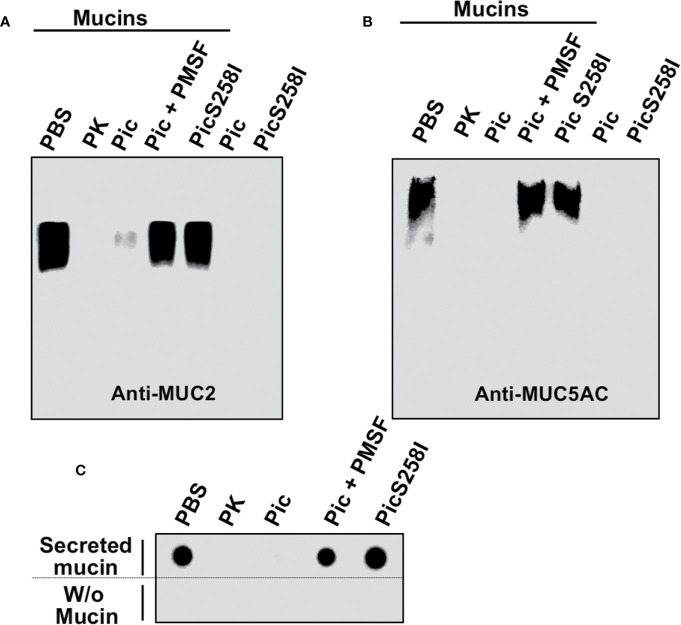
Proteolytic degradation of MUC2 and MUC5AC by Pic. **(A, B)** Pic native, but not Pic inactive in its serine protease motif, degrades MUC2 **(A)** and MUC5AC **(B)**. LS174T cells were incubated with 2 µg/ml of Pic, PMSF-preincubated Pic or PicS258I, at 37°C for 2 h. Preparations were run in SDS-agarose and analyzed by Western blot using anti-MUC2 Ccp58 or anti-MUC5AC 45M1 antibodies; PBS was used as a negative control and Proteinase K as a positive control. **(C)** Pic degrades non-denatured mucins. Mucin secreted by LS174T cells were incubated with 2 µg/ml of Pic, PMSF-preincubated Pic or PicS258I, at 37°C for 2 h. Samples were dropped on a nitrocellulose membrane and analyzed by Dot-blot using the anti-MUC5AC antibody (45M1).

To further characterize the proteolytic activity of Pic on mucins, LS174T cell lysates were incubated with Pic, PMSF-preincubated Pic, or PicS258I for degradation assays. Proteinase K was used as a positive control, and PBS (vehicle) was used as a negative control. Following a time-course incubation, samples were separated by SDS-agarose gel electrophoresis and analyzed by Western blotting using anti-MUC5AC (45M1) or anti-MUC2 (Ccp58) antibodies (**Figures 3A, B**). Both mucins, MUC5AC and MUC2, were detected by the respective antibodies, but the MUC2 band was more intense than the MUC5AC band. Remarkably, in mucins from LS174T cell lysates incubated with native Pic, the antibodies were unable to detect MUC2 or MUC5AC, and the lack of detection of these mucins was similar to that of samples treated with proteinase K, the positive control for proteolysis. In contrast, PMSF-preincubated Pic and the serine protease motif mutant were unable to degrade MUC2 and MUC5AC, and the respective antibodies detected both mucin bands by Western blotting. To further understand the extension of the MUC2 degradation by Pic, we used another anti-MUC2 (H-300) antibody, which was unable to detected MUC2 in samples treated with Pic, but it did in samples treated with Pic inactivated in the serine protease motif (**Figure S3C**). This polyclonal antibody recognizes an epitope in the C-terminal portion (4,880–5,179 aa) instead the anti-MUC2 (Ccp58) that recognizes a repeat sequence GTQTP in the centrally localized PTS domains. To verify these data, we purified the total mucins secreted by LS174T cells and incubated these mucins with Pic, PMSF-preincubated Pic, or PicS258I in degradation assays and then MUC5AC was immunodetected by dot-blotting. Anti-MUC5AC (45M1) was able to detect MUC5AC secreted by untreated human goblet-like cells that had been dropped under nondenaturing conditions onto nitrocellulose membranes. Fascinatingly, when secreted mucins were incubated with native Pic for 2 h, the anti-MUC5AC antibody was unable to detect MUC5AC, indicating the degradation of this mucin by Pic; a similar result was obtained with proteinase K. In contrast, PMSF prevented MUC5AC degradation by Pic and the PicS258I mutant was unable to degrade MUC5AC, as revealed by an antibody signal similar to that of PBS treatment (**Figure 3C**). Taken together, these data indicate that Pic is a potent protease that is able to degrade the mucins secreted by goblet cells and that the serine protease motif is essential for this proteolytic process.

**Figure 4:**
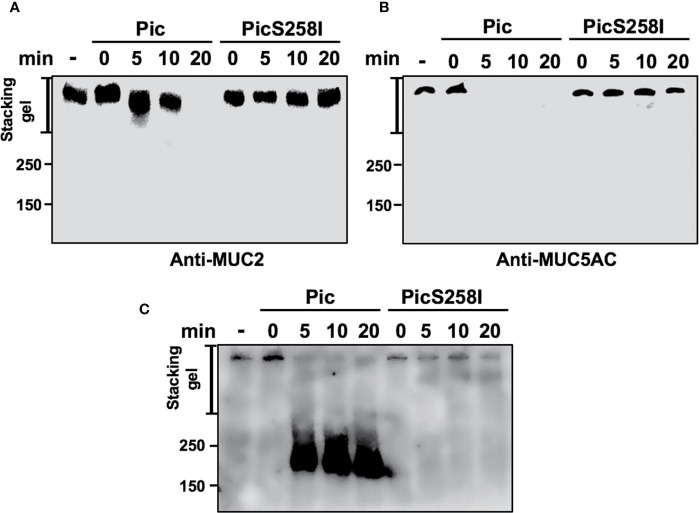
Pic cleaves mucins in their C-terminal domain. **(A, B)** Pic degrades more efficiently MUC5AC than MUC2. Soluble fractions from lysates of LS174T cells were incubated 2 µg/ml of Pic **(A)** or PicS258I **(B)** at different times. Mucin were run in SDS-PAGE and analyzed by Western blot using anti-MUC2 Ccp58 or anti-MUC5AC 45M1 antibodies. **(C)** Detection of the degradation subproducts from MUC5AC. MUC5AC degradation was analyzed by Western blot using an anti-MUC5AC H-160 antibody to detect the N-terminal domain (1,214–1,373 aa), since the anti-MUC5AC 45M1 is directed to the C-terminal domain.

To determine the degradation kinetics and detect the of degradation subproducts from these mucins, LS174T cell lysates were incubated with native Pic or PicS258I for different periods of time (0, 5, 10, and 20 min), and MUC2 and MUC5AC were separated by SDS-PAGE and detected by Western blotting using anti-MU2 (Ccp58) and anti-MUC5AC (45M1) antibodies. The MUC2 protein immunoblot signal was undetectable at 20 min of incubation with Pic (**Figure 4A**), whereas the MUC5AC signal disappeared more rapidly and was undetectable at 5 min of incubation (**Figure 4B**). In contrast, PicS258I was unable to degrade MUC2 or MUC5AC at any tested time. Interestingly, no degradation subproducts were detected from these two mucins, suggesting that the degradation process is very thorough or that the epitopes detected by the antibodies used are degraded first. To test the latter possibility, we used another anti-MUC5AC antibody, a polyclonal antibody (H-160) that recognizes the N-terminal part of MUC5AC (aa 1,214 to 1,373) instead of the monoclonal anti-MUC5AC (45M1) antibody, which recognizes the C-terminus of this protein (aa 4,627 to 4,731). Thus, the PVDF membrane used in **Figure 4B** was stripped and probed with this polyclonal antibody. As expected, the polyclonal anti-MUC5AC antibody recognizing the N-terminal domain of MUC5AC was able to detect an intense and smeared band corresponding to diverse subproducts of degradation between 250 and 180 kDa, as shown in the separating gel (**Figure 4C**). These MUC5AC degradation subproducts were detected starting at 5 min of incubation with Pic and remained stable throughout the time course until 20 min. These data obtained from SDS-PAGE gels were similar to those obtained in SDS-agarose gels electrophoresis (**Figures S3A, B, D**). These data clearly show that Pic has strong and efficient proteolytic activity on human mucins secreted by goblet cells through its serine protease motif and that its cleavage site is localized in the C-terminal domain.

### Pic Did not Induce Mucus Secretion Through the Cytokine Pathway

It has been reported that different cytokines such as TNF-α, IL-6, and IL-13, increase mucin secretion in goblet cells (30, 31). To determine whether Pic increases cytokine secretion that leads to mucin hypersecretion in an autocrine fashion, LS174T cells were incubated with Pic or PicS258I (5 µg/ml) for 4 h, and the supernatants were collected for cytokine quantification using a human inflammatory cytokine detection kit (cytometric bead array kit) to measure IL-1β, IL-6, IL-8, IL-10 IL-12p70, and TNF-α. Pic or PicS258I did not cause an increase in the secretion of TNF-α, IL-6, IL-1β, or the other tested cytokines, except for IL-8. Pic or PicS258I slightly increased IL-8 secretion levels to 0.15 ng/ml compared with those induced by stimulation with phorbol ester (PMA) as a positive control, which was of 1.7 ng/ml (**Figure S4**). These data strongly suggest that Pic does not increase mucins secretion through these cytokines in an autocrine manner. Although Pic slightly increased IL-8 secretion, this cytokine has not been shown to be involved in the hypersecretion of mucins by intestinal goblet cells.

### Pic Induces the Intracellular Calcium Pathway to Increase Mucus Secretion

**Figure 5:**
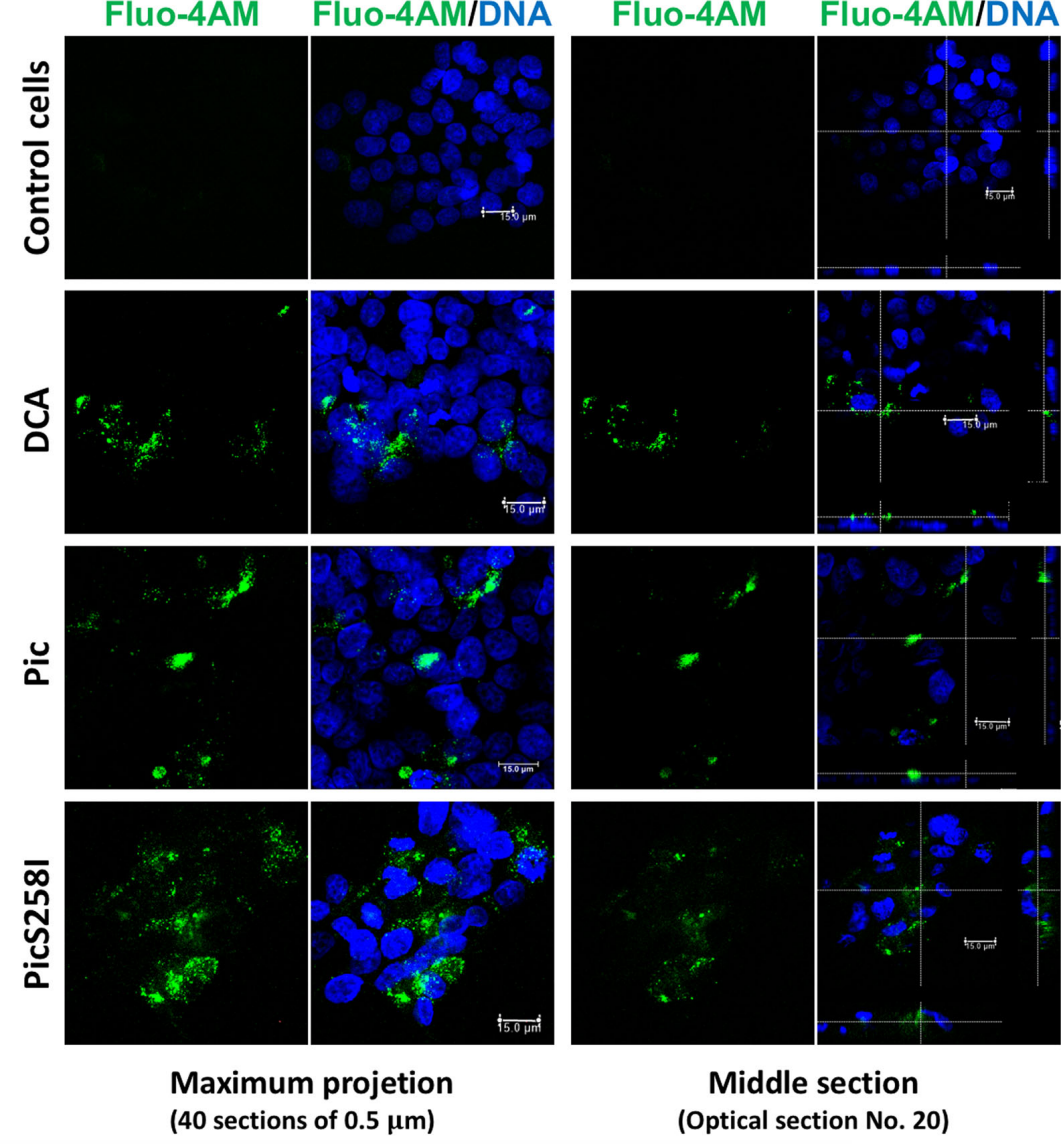
Pic induces an increase of cytosolic calcium in goblet-like cells. LS174T cells were incubated with a calcium indicator, Fluo-4-AM (2 µM) (green). Then, loaded cells were incubated with 5 µg/ml of Pic, PicS258I or 0.125 mM DCA, at 25°C for 4 h. Cells were stained with DAPI to stain nuclei (blue) and analyzed by confocal microscopy, Leica TCS SP8 (Leica Microsystems).

To explore the mechanism by which Pic induces mucus hypersecretion, we first explored an important cellular messenger in mucin secretion induced by goblet cells, the intracellular calcium. Calcium plays a critical role in the signals leading to mucin secretion. To examine this possibility, LS174T cells were first loaded with the calcium-binding probe Fluo-4-AM (green) and then incubated with Pic or PicS258I for 4 h; DCA was used as a positive control. The treated cells were fixed, and the nuclei were stained with TO-PRO-3 and analyzed by confocal microscopy. Pic or PicS258I induced an increase in the fluorescent green signal inside LS174T cells due to the increase in intracellular calcium bound to Fluo-4-AM, similar to the signal intensity observed in cells treated with DCA. Untreated cells did not show an increase in intracellular calcium (**Figure 5**). These data show that both native Pic and PicS258I increase the intracellular calcium as a pathway to increase mucin hypersecretion.

**Figure 6:**
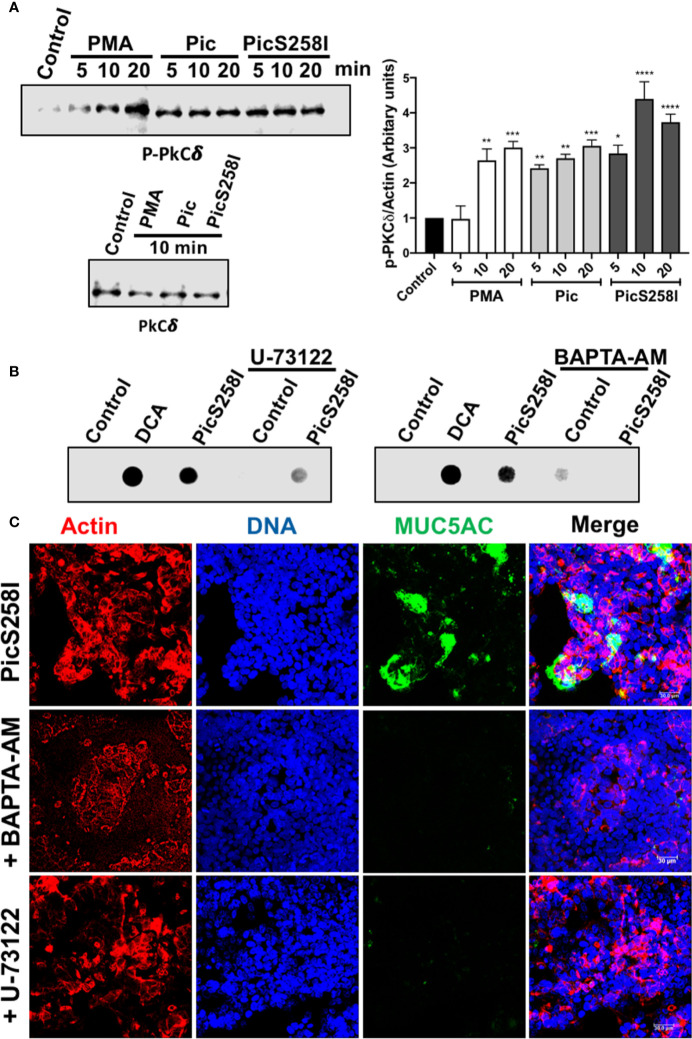
Pic induces PKC activation and calcium release through PLC pathway in goblet-like cells. **(A)** PKC activation. LS174T cells were incubated with 5 µg/ml of Pic, PicS258I, or 2 µM PMA for different times. Cell lysates were analyzed by Western blot using anti-p-PKCδ at different times (5, 10, and 20 min) or anti-PKCδ-total (at 10 min) (left panels). A densitometric analysis of Western blot assays was performed (right panel). Data are shown as mean ± SEM of at least 3 independent experiments. Statistical analysis was performed using one-way ANOVA with Dunnet’s *post hoc* test, *p < 0.05%, **p < 0.01%, ***p < 0.001%, ****p < 0.0001%. **(B, C)** inhibition of MUC5AC secretion using a PLC inhibitor, U73122, or the calcium chelator, BAPTA-AM. LS174T cells were incubated with either U73122 or BAPTA-AM for 30 min and then incubated with 5 µg/ml of Pic or PicS258I for 4 h, in the presence of the inhibitor. Then supernatants of treated cell were analyzed by Dot-blot using and anti-MUC5AC antibody followed by a secondary antibody coupled to HRP **(B)**. While, cells were analyzed by confocal microscopy immunostaining with the anti-MUC5AC followed by a secondary antibody coupled to fluorescein (green) **(C)**. Cells were stained with rhodamine-phalloidin for detecting actin and TO-PRO-3 for nuclei.

Since the intracellular calcium could be result from the DAG and IP_3_ production to target Munc13-2 that is activated by DAG and Syt2 (synaptotagmin-2) that is activated by calcium (32, 33), we also analyzed the role of PLC, which is upstream of DAG and IP_3_, on the secretion of MUC5AC by using the PLC inhibitor U-73122. To avoid mucin degradation, LS174T cells were treated with PicS258I with and without preincubation with U-73122 for 30 min and MUC5AC secretion was measured in the supernatants by dot-blotting using an anti-MUC5AC antibody. DCA, the positive control, and PicS258I increased MUC5AC secretion as determined as strong dots, while in LS174T cells treated with the inhibitor, this MUC5AC secretion by PicS258I was clearly decreased (**Figure 6B**, left panel). As the PLC pathway activation results in an intracellular increase in calcium, leading to mucin secretion, a calcium chelator was used to block the increase in intercellular calcium. Cells were treated as mentioned above, but BAPTA-AM was used instead of U-73122, and the treated cells were also analyzed by dot-blotting. BAPTA-AM chelated the intracellular calcium induced by PicS258I and thus MUC5AC secretion, as observed by the lack of a dot for this mucin, whereas in cells treated with PicS258I but without BAPTA-AM, MUC5AC was clearly detected as a dot (**Figure 6B**, right panel).

To confirm these results, we analyzed the inhibition of MUC5AC secretion in the presence of a PLC inhibitor and calcium chelator by confocal microscopy. The experiments were similar to dot-blot assays but cells were analyzed by immunostaining for MUC5AC rather than supernatants. As expected, confocal microscopy showed that PicS258I increased MUC5AC secretion (green) in goblet-like cells (nuclei in blue and actin in red). The PLC inhibitor U-73122 or the calcium chelator, BAPTA-AM inhibited the Pic-induced MUC5AC secretion, and no green signal from the anti-MUC5AC antibody was detected in these treated cells (**Figure 6C**).

In addition to Pic-mediated hypersecretion of mucins through the intracellular calcium as showed above, it is known that activation of PKC further modulates secretory function (34, 35). To further explore this pathway by measuring PKCδ activation, LS174T cells were treated with 5 µg/ml of Pic, PicS258I, or PMA (2 µM) at different time intervals. Cell lysates were analyzed by Western blotting using anti-PKCδ and anti-p-PKCδ (anti-phosphorylated PKCδ). Both Pic and PicS258I induced PKCδ activation by increasing PKCδ phosphorylation beginning at 5 min of treatment, and this increase was also detected at 10 and 20 min of treatment (**Figure 6A**) and the densitometric analysis showed that PKCδ activation was significantly statistical compared with control cells (**Figure 6A**). PMA increased PKCδ phosphorylation in a time-dependent manner, reaching similar activation levels as that of Pic at 10 min but a higher activation at 20 min. The total amount of PKCδ protein was unchanged by all treatments (**Figure 6A**).

### Pic Uses Mucus Secretion as a Carbon Source and Mucus Degradation to Pass Through the Mucus Layer

**Figure 7:**
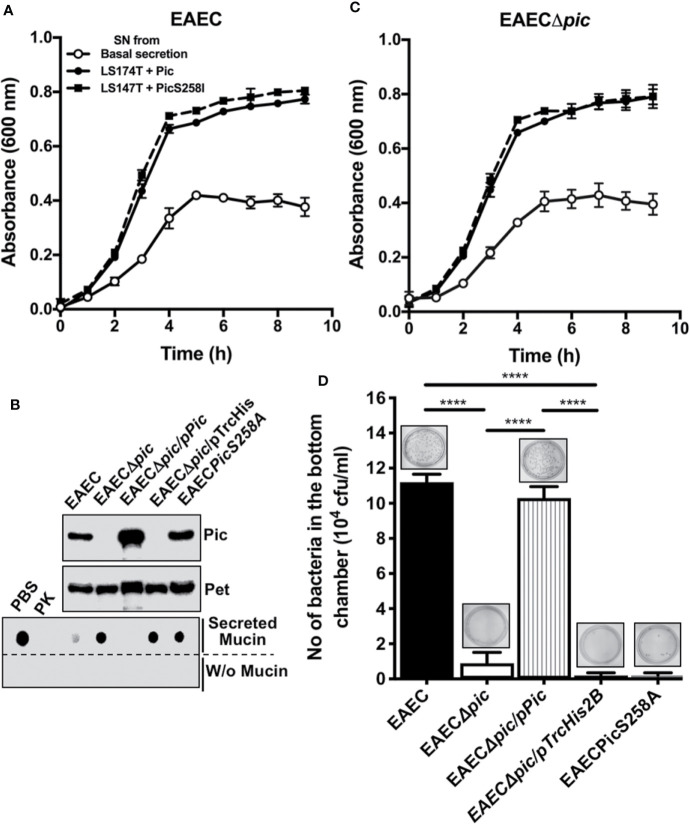
EAEC, but not a *pic* mutant, is able to efficiently penetrate the mucin barrier and both strains use the secreted mucins as carbon source for their growth. **(A)** Growth curves of EAEC and EAECΔ*pic* using conditioned media from LS174T cells. Supernatants (conditioned media) from untreated LS174T cells (basal secretion) or from LS174T cells stimulated with 5 µg/ml of Pic or PicS258I (LS174T + Pic and LS174T + PicS258I, respectively) at 37°C for 4 h were used to grow EAEC and EAECΔ*pic* in an 8 h kinetics. **(B)** Pic expression and proteolytic activity of EAEC variants. TCA-precipitated supernatants from EAEC, EAECΔ*pic*, EAECΔ*pic*/p*Pic*, EAECΔ*pic*/pTrcHis2B, EAEC*pic*S258A were analyzed by Western blot using an anti-Pic antibody (upper panel) or an anti-Pet antibody (middle panel). (Lower panel) Supernatants from the last different EAEC variants were individually incubated with secreted mucins from LS174T cells (0.5 µg) at 37°C for 2 h (pH 7.2) or in absence of mucins and analyzed by Western blot using anti-MUC5AC antibodies (45M1). **(C)** EAEC use the proteolytic activity of Pic to penetrate the mucus layer. Secreted mucins from LS174T cells (5 µg/200 µl) were layered in the insert of Transwell chambers (diameter of 1.8 mm and pores of 3 µm). The different EAEC variants (20 × 10^6^ CFUs) were inoculated on the mucin-containing insert (upper chamber), while LB medium was placed in the lower chamber and the whole Transwell was incubated at 37°C for 2 h. Then, inserts were removed and the media of the lower chamber were plated on LB-agar plates to quantify those bacteria (CFUs) that passed through the mucin layer. Data are representative of 3 independent experiments. Statistical analysis was performed using one-way ANOVA with Dunnet’s *post hoc* test, ****p < 0.0001%.

To further understand the dual activity of Pic during EAEC infection, we first investigated the role of Pic-induced mucus secretion and mucus availability on EAEC growth by incubating EAEC with conditioned media from LS174T cells treated with Pic (degraded mucins) and LS174T cells treated with PicS258I (undegraded mucins). EAEC was also grown in basal mucin secretion from LS174T cells as a control. Growth curves showed that EAEC grew better in conditioned media from Pic- or PicS258I-treated LS174T cells than in medium containing basal mucin secretion from untreated LS174T cells (**Figure 7A**). There was no significant difference between EAEC growth in conditioned medium from LS174T cells treated with Pic or PicS258I, suggesting that EAEC uses whole or partially degraded mucins as a carbon source. To corroborate these results, similar experiments were performed, but now an isogenic mutant of *pic* (EAECΔ*pic*) was grown in the conditioned media described above. EAECΔ*pic* grew in a very similar way as the wild-type EAEC in conditioned media from LS174T cells treated with either Pic or PicS258I (**Figure 7C**). EAEC growth in media containing mucins induced by either Pic or PicS258I was significantly better than that in media containing a basal secretion of these mucins (**Figure 7A**).

To determine whether Pic is used by EAEC to penetrate the mucin layer, several EAEC variants were used: EAEC, EAECΔ*pic*, EAECΔ*pic/*p*Pic*, EAECΔ*pic/*pTrcHis2B (the vector), or a chromosomal mutant in the serine protease motif (EAECPicS258A). Before the analysis of bacterial penetration of the mucus layer, the ability of each EAEC variant to induce mucin secretion and degradation was evaluated. Supernatants from the EAEC variants were precipitated with 20% TCA and analyzed by Western blotting using anti-Pic antibodies and an anti-Pet antibody as a control. As expected, all the EAEC variants secreted the Pet protein, whereas Pic was secreted and detected in supernatants from the EAEC, EAECΔ*pic/*p*Pic*, and EAECPicS258A variants but not in those from EAECΔ*pic* and EAECΔ*pic/*pTrcHis2B variants (**Figure 7B**). Concentrated supernatants from the EAEC variants were incubated with mucins secreted by LS174T cells and showed that Pic secreted by EAEC and EAECΔ*pic/*p*Pic* were able to degrade MUC5AC, as shown by dot blot using anti-MUC5AC antibodies, similar to those treated with Proteinase K as a control. However, the Pic protein secreted by EAECPicS258A was unable to degrade MUC5AC (similar to PBS used as a control) as well as those strains unable to secrete Pic, EAECΔ*pic* and EAECΔ*pic/*pTrcHis2B (**Figure 7B**).

Once tested the ability of each EAEC variant to degrade MUC5AC was tested, all variants were used for mucus layer penetration experiments. A layer of mucins secreted by LS174T was formed in a Transwell device, the different EAEC variants were placed in the upper chamber at 20 × 10^6^ CFU, and LB medium was added to the lower chamber. After incubation for 2 h at 37°C, the upper chamber was removed, and the LB medium was used to quantify the CFUs that passed through the mucin layer. Clearly, EAEC efficiently penetrated the mucus layer (1.1 × 10^5^ CFU) compared with the *pic* mutant EAECΔ*pic* (1 × 10^4^ CFU) (**Figure 7D**). Furthermore, EAECPicS258A, a Pic-producing strain with a mutation in the serine protease motif, was unable to pass through the mucin layer. As expected, the *pic* mutant that was complemented with the *pic* gene (EAECΔ*pic/*p*Pic*) was able to pass through the mucin layer at slightly higher levels than the wild-type EAEC. While the variant complemented with the empty vector (EAECΔ*pic/*pTrcHis2B) was unable to pass through the mucin layer (**Figure 7D**). These data show that Pic is relevant as a serine protease that degrades mucus to allow EAEC penetrate the mucus layer, and EAEC uses the mucus hypersecretion as a carbon sources, regardless of whether the mucins are degraded.

## Discussion

A hallmark of EAEC infection is the formation of a biofilm, which is composed of bacteria immersed in the mucus layer within in the intestines of patients; however, the mechanism leading to this exacerbated mucus secretion induced by EAEC is not fully understood. Here, we further studied the mechanism underlying the hypersecretion of mucus induced by Pic. Remarkably, Pic was able to directly induce the hypersecretion of mucus by goblet cells. Interestingly, Pic exhibited strong degradation activity on secreted mucins. These activities were independent since a mutation in the serine protease motif (PicS258I) abolished catalytic degradation of mucin but not the segretagogue activity of mucin. In fact, the induction of mucus secretion by the known secretagogue, deoxycholic acid (DCA), results in synergistic activity when was simultaneously used with the mutant PicS258I, but these mucins were also degraded when DCA was simultaneously used with the native Pic. Preincubation of Pic with a serine protease inhibitor exhibited similar effects as those induced by the catalytic motif mutant. MUC2 and MUC5AC are both degraded by Pic, but not by either the serine protease motif mutant of Pic or Pic that was preincubated with the serine protease inhibitor. Pic was able to cleave MUC5AC in the C-terminal portion, leaving degradation subproducts of 180–250 kDa corresponding to the N-terminal part of MUC5AC. Excitingly, Pic stimulated the rapid secretion of mucin directly by goblet-like cells by activating the intracellular calcium pathway, resulting in the PLC signal transduction pathway leading to DAG and IP_3_ production; the latter functions as a calcium signaling pathway second messenger. Finally, the dual activities of Pic as a mucus secretagogue and mucinase are relevant for the use of mucus as a carbon source and for the ability to penetrate the mucus layer by EAEC to reach the epithelial cells.

Pic-induced mucus hypersecretion directly in the goblet-like cells suggests that no other cell types are required, such as enterocytes, enteroendocrine cells or Paneth cells. These resident cells of the intestinal mucosa could secrete bioactive factors, such as EGF by the activating EGFR and the MAPK pathway (36, 37), prostaglandin, which is reported to be a mucin secretagogue (38), through activation of CREB/ATF1 (39), as well as cytokines such as IL-4, IL-13 and TNF-α (31), IL-6 (40) or IL-1β (41) through NF-κB activation mediated by MAPK, to indirectly activate goblet cells. Interestingly, Pic-induced mucus hypersecretion did not appear to involve an autocrine mechanism through cytokine secretion; incubation of goblet-like cells with Pic did not induce TNF-α, IL-6, or IL-1β secretion. However, during EAEC infection, proinflammatory cytokines could enhance mucus secretion through enterocytes, since among the *E. coli* pathotypes, EAEC is the best inducer of TNF-α secretion in epithelial cells and induces the secretion of TNF-α, IL-6, or IL-1β by macrophages (42). Interestingly, Pic has this dual activity; therefore, the hypersecretion of mucus may be not be properly detected since the secreted mucus can be degraded by the protease activity of Pic, as shown in the present study. Thus, in rat ileal loops inoculated with Pic from EAEC, *S. flexneri* and UPEC, abundant mucus was detected in the intestinal lumen (21), while in mice infected with PicC from *C. rodentium*, no differences were detected between the wild type and the isogenic *picC* mutant (43).

Here we have shown that in addition to its secretagogue activity, Pic is also able to efficiently degrade the secretory gel-forming mucins, mainly MUC2 and MUC5AC. According to our findings, Pic cleaves MUC2 and MUC5AC at the C-terminus, and the similarity of MUC5AC in the number and positions of the cysteine residues in the C-terminus with MUC2 is greater than 90% (44). However, MUC2 and MUC5AC do not heterodimerize when present in the same cell (2), suggesting that the sequences outside the “cystine knot” motif are different and also involved in the specificity of dimer formation. Regarding to the antibody recognition, Pic treatment eliminated the epitope GTQTP, which is recognized by the anti-MUC2 antibody (Ccp58). Interestingly, this epitope is a highly repetitive sequence in the PTS domains [interrupted by cysteine domains (CysD)]. This epitope occurs 99 times in these domains from the amino acid 1,915 to the amino acid 4,173. Additionally, the rabbit polyclonal anti-MUC2 (H-300), which recognizes a C-terminal portion (amino acids 4,880–5,179), also showed MUC2 degradation. These data strongly suggest that these epitopes were proteolytically degraded by Pic and that a large part of MUC2 was degraded. Unfortunately, the GTQTP epitope is lacking in MUC5AC, which avoids corroborating this effect on MUC5AC. However, Pic treatment eliminated the epitope recognized by the anti-MUC5AC antibody (45M1), which is thought to recognize a sequence in the last C-terminal CysD domain of this mucin (4,627–4,731 aa) and does not recognize this epitope in reducing conditions (45). The use of the polyclonal anti-MUC5AC antibody (H160), which recognizes epitopes in the amino acids 1,214–1,376, allowed to detect subproducts of MUC5AC degradation that were able to enter into the polyacrylamide gel, showing a smeared band from 180 to 250 kDa. Due to its size, the putative 180 kDa fragment would also exclude the large PTS domains. These data strongly suggest that Pic is degrading a large part of the MUC2 and MUC5AC polypeptides and suggest that the cleavage site is between the end of the nonglycosylated N-terminus and the beginning of the PTS domains (around aa 1,680–1,915). Degradation of this large part of MUC5AC or MUC2 mainly to the C-terminal portion is critical for gel-forming mucins, since these mucins form dimers in the far C-terminal end, by the cysteine-knot domain (6). Two other pathogenic enzymes, a cysteine protease from *E. histolytica* (EhCP5) and a bacterial cysteine protease from *Porphyromonas gingivalis* (RgpB), are capable of cleaving the MUC2 protein core; EhCP5 cleaves the SIIRT↓TGLR sequence and one amino acid away, RgpB cleaves SIIR↓TTGLR. It is clear that *P. gingivalis* cleaves the MUC2 C-terminal region, whereas the N-terminal region is unaffected (46). Again, the less glycosylated N- and C-terminal domains are stabilized by numerous disulfide bonds and make the MUC2 C-terminally dimeric and N-terminally trimeric (7), and a cleavage in the C-terminal domain of MUC5AC or MUC2 by EhCP5, RgpB, or Pic destroys the large net-like sheets of the gel-forming mucins. Furthermore, the mucus layer normally acts as a barrier that prevents the bacteria from reaching epithelial cells and thus limits the direct contact between the host and the bacteria (47). However, the cleavage of MUC2 or MUC5AC mucins at the C-terminus causes the mucus polymer to dissolve. In the case of *E. histolytica*, this cleavage disrupts the MUC2 polymer and allows the amoeba to penetrate the inner mucus layer (48). Here, we found that Pic-secreting EAEC are able to efficiently penetrate the mucus layer, but not variants with mutations in the serine protease motif of Pic. Interestingly, the cleavage of mucins by Pic did not favor EAEC growth in comparison with that of conditioned medium from LS174T cells treated with PicS258I, since both treatments increased EAEC growth to similar levels and were clearly higher than of conditioned medium from untreated LS174T cells (basal secretion). These data suggest that mucin degradation by Pic does not enhance the efficiency of O-linked oligosaccharide degradation for glycan-degrading enzymes; the intestinal bacteria are very efficient in degrading the mucus oligosaccharides, and up to 40% of the bacterial genomes encode glycan-degrading enzymes (49). Thus, similar to commensal colonic bacteria, EAEC can efficiently utilize the monosaccharides released by the bacterial hydrolases and transported into the bacteria by specific transporters (49, 50), independently of whether the apomucin is cleaved.

The extracellular ligands and signal transduction pathways that regulate mucin secretion have been studied for a long time (51). ATP is the best studied ligand and acts on the P2Y2 receptors to activate Gq and PLC-1, leading to generation of the second messengers IP_3_ and DAG. However, many pathogens can facilitate the intestinal colonization through the regulation of goblet cells and mucin expression (52). For instance, *Vibrio cholerae* stimulates massive mucin secretion by increasing the intracellular cAMP (53), which leads to the activation of cAMP response element-binding protein (CREB) (54). *E. histolytica* stimulates potent mucin secretion by a contact-dependent mechanism involving PKC (55). Other parasites, such as *Nippostrongylus brasiliensis*, *Trichinella spiralis* and *Trichuris muris*, induce cytokine production by immune cells to significantly increases mucus production (56–58). Here, we used LS174T cell cultures and showed that Pic did not increase mucin secretion by paracrine or autocrine stimulation through cytokines. Instead, Pic induces mucus hypersecretion by the intracellular calcium pathway. This cytoplasmic calcium must be from intracellular stores, in contrast to excitable cells in which calcium enters the cytoplasm from outside the cell through voltage-gated channels (51). The Pic-induced intracellular calcium increase suggests the involvement of PLC in the signaling pathway. PLC catalyzes PIP_2_ conversion to IP_3_ and DAG; IP_3_ is the major contributor to intracellular calcium release (59) and DAG targets Munc13 (32). Accordingly, we found that in LS174T cells, Pic-induced mucin secretion was PLC-dependent (upstream of IP_3_ and DAG), as shown by experiments with the PLC inhibitor U73122. Additionally, we found that PKCδ, a mucus hypersecretion enhancer, was activated by Pic treatment and it is known that PKCδ activation enhances mucin secretion (34, 35). Interestingly, mucin hypersecretion is induced by Pic independently of the serine protease motif, indicating that, unlike its mucinolytic activity, the proteolytic activity is not required. Mucus hypersecretion is a common feature in chronic airway diseases, and serine proteases play a critical role in this process (60, 61). PARs (protease activated receptors) are G-protein-coupled receptors that are activated by the cleavage of their N-terminal domain (62), and this process occurs upstream of PLC, leading to intracellular calcium release and mucus secretion. Thus, more studies are required to understand how Pic induces the extracellular signaling to trigger the PLC/IP_3_-DAG/intracellular calcium pathway.

## Data Availability Statement

The raw data supporting the conclusions of this article will be made available by the authors, without undue reservation.

## Author Contributions

FN-G and FF-S participated in the design of the study, data analysis, and writing of the manuscript. FF-S carried out most of the experiments. JS-V and LC-D assisted with several key experiments. All authors contributed to the article and approved the submitted version.

## Funding

This work was supported by a grant from Consejo Nacional de Ciencia y Tecnología (CONACYT 221130) to FN-G.

## Conflict of Interest

The authors declare that the research was conducted in the absence of any commercial or financial relationships that could be construed as a potential conflict of interest.

## Supplementary Material

Supplementary Figure 1 Deoxycholic acid (DCA) induces secretion of both MUC5AC and MUC2 on goblet cell-like, LS174T cells. LS174T cells were stimulated with different concentrations of DCA at 37°C for 4 h. Untreated cells were used as a positive control, basal secretion from LS174T cells. After stimulations, supernatants were recovered and MUC5AC or MUC2 were detected by ELISA using either monoclonal anti-MUC5AC **(A)** or anti-MUC2 **(B)** antibodies and developed with a secondary antibody coupled to HRP. Data are shown as mean ± SEM of at least 3 independent experiments. Statistical analysis was performed using one-way ANOVA with Dunnet’s *post hoc* test, **p* < 0.05%, ***p* < 0.01%, ****p* < 0.001%, *****p* < 0.0001%.

Supplementary Figure 2 Pic does not affect LS174T cell viability. LS174T cells (25,000) were grown in 98-well plates at 37°C for 72 h, until confluency. Then, cells were kept 17 h in DMEM medium without FBS. LS174T cells were stimulated with 5 µg/ml of Pic, or PicS258I, or 0.125 mM DCA, at 37°C for 4 h. Then, cells were incubated with 0.5 mg/ml MTT (3-[4,5-dimethylthiazol-2-yl]-2,5 diphenyl tetrazolium bromide) in DMEM medium with 10% FBS at 37°C and 5% CO_2_ for 30 min. Washed cells were lysed by adding DMSO (Dimethyl sulfoxide) and samples were read at an optical density of 630 nm in a iMark Microplate Absorbance Reader. Statistical analysis was performed using one-way ANOVA with Dunnet’s *post hoc* test, *****p* < 0.0001%.

Supplementary Figure 3 MUC2 and MUC5AC degradation by Pic analyzed in SDS-agarose gels electrophoresis and Western blot using different antibodies. **(A**, **B)** Pic degrades more efficiently MUC5AC than MUC2. Soluble fractions from lysates of LS174T cells were incubated 2 µg/ml of Pic (A) or PicS258I (B) at different times. Mucin were electrophoresed in SDS-agarose gels and analyzed by Western blot using anti-MUC2 (Ccp58) or anti-MUC5AC (45M1) antibodies. **(C**, **D)** Detection of different MUC2 and MUC5AC epitopes using anti-MUC2 (H-300) and anti-MUC5AC (H-160) antibodies. (C) MUC2 degradation was analyzed by Western blot using an anti-MUC2 H-300 antibody to detect the C-terminal domain (1,214–1,373 aa). (D) MUC5AC degradation was analyzed by Western blot using an anti-MUC5AC H-160 antibody to detect the N-terminal domain (4,880–5,179 aa).

Supplementary Figure 4 Pic does not increase secretion of IL-1β, TNF-α, IL-6, but a slightly the IL-8 secretion. LS174T cells (500,000) were grown in 30 mm plates at 37°C for 48 h. Then, cells were kept 17 h in DMEM medium without FBS. LS174T cells were stimulated with 5 µg/ml of Pic, or PicS258I at 37°C for 4 h. After stimulations, supernatants were recovered and analyzed using a human inflammatory cytokines CBA kit (BD Biosciences). Phorbol 12-myristate 13-acetate (PMA, 2 µM) was used as a positive control and Pet protein was used as a negative control. Data were acquired in a BD FACS Fortessa flow cytometer. Statistical analysis was performed using one-way ANOVA with Dunnet’s *post hoc* test, ****p* < 0.001%, *****p* < 0.0001%.
