# Supplementary figures and images for: Pic Protein From Enteroaggregative E. coli Induces Different Mechanisms for Its Dual Activity as a Mucus Secretagogue and a Mucinase
Source: Front Immunol. 2020 Nov 17;11:564953. doi: 10.3389/fimmu.2020.564953 (PMC7705071; doi:10.3389/fimmu.2020.564953)

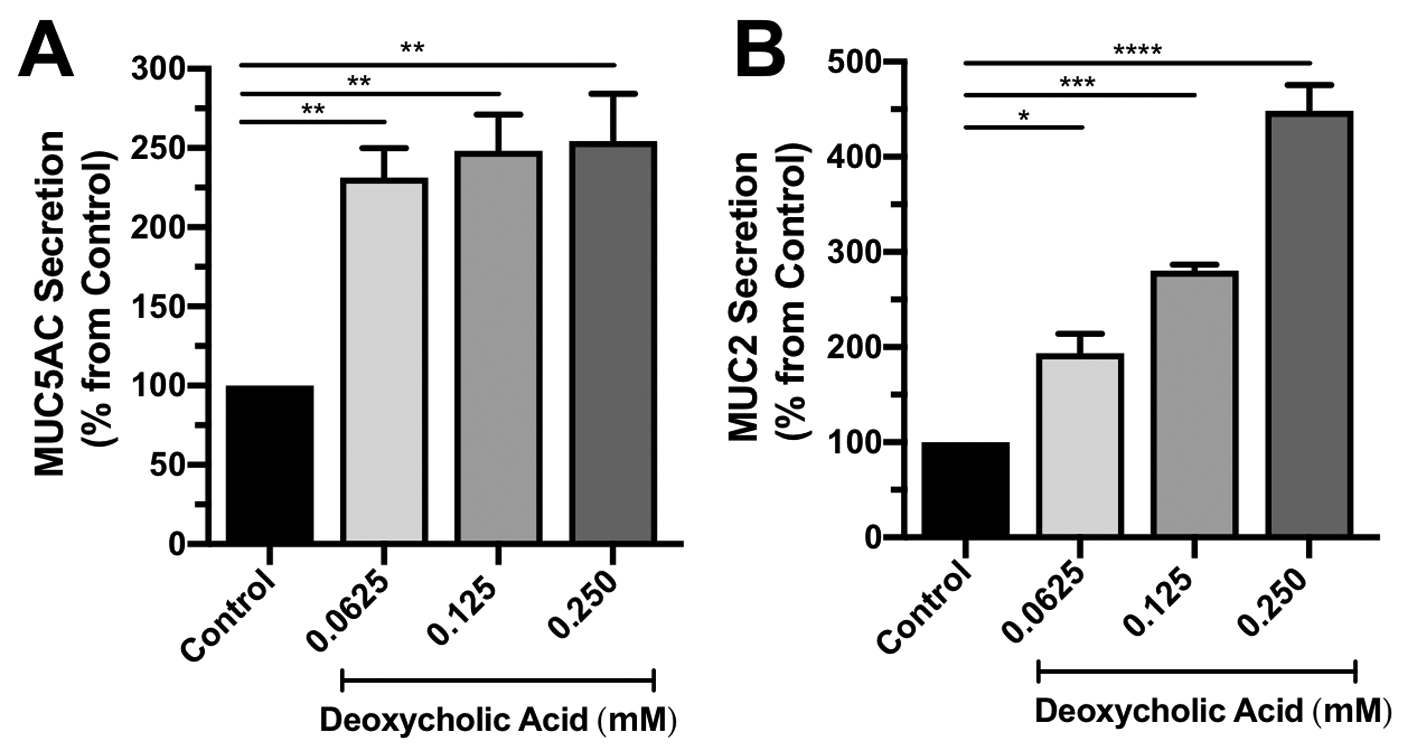

Supplement: Supplementary Figure 1 — Deoxycholic acid (DCA) induces secretion of both MUC5AC and MUC2 on goblet cell-like, LS174T cells. LS174T cells were stimulated with different concentrations of DCA at 37°C for 4 h. Untreated cells were used as a positive control, basal secretion from LS174T cells. After stimulations, supernatants were recovered and MUC5AC or MUC2 were detected by ELISA using either monoclonal anti-MUC5AC (A) or anti-MUC2 (B) antibodies and developed with a secondary antibody coupled to HRP. Data are shown as mean ± SEM of at least 3 independent experiments. Statistical analysis was performed using one-way ANOVA with Dunnet’s post hoc test, *p < 0.05%, **p < 0.01%, ***p < 0.001%, ****p < 0.0001%. [file Image_1.tif]

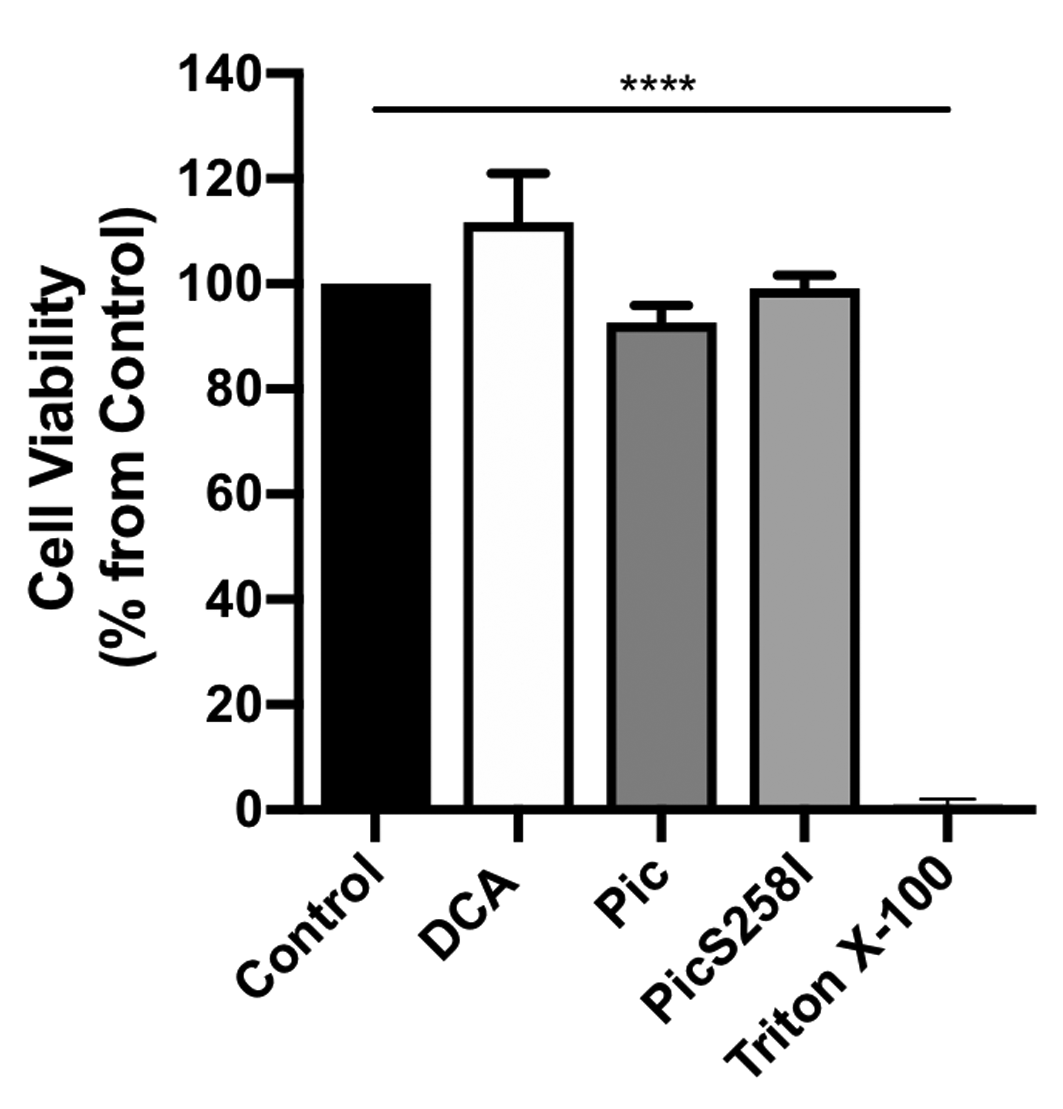

Supplement: Supplementary Figure 2 — Pic does not affect LS174T cell viability. LS174T cells (25,000) were grown in 98-well plates at 37°C for 72 h, until confluency. Then, cells were kept 17 h in DMEM medium without FBS. LS174T cells were stimulated with 5 µg/ml of Pic, or PicS258I, or 0.125 mM DCA, at 37°C for 4 h. Then, cells were incubated with 0.5 mg/ml MTT (3-[4,5-dimethylthiazol-2-yl]-2,5 diphenyl tetrazolium bromide) in DMEM medium with 10% FBS at 37°C and 5% CO2 for 30 min. Washed cells were lysed by adding DMSO (Dimethyl sulfoxide) and samples were read at an optical density of 630 nm in a iMark Microplate Absorbance Reader. Statistical analysis was performed using one-way ANOVA with Dunnet’s post hoc test, ****p < 0.0001%. [file Image_2.tif]

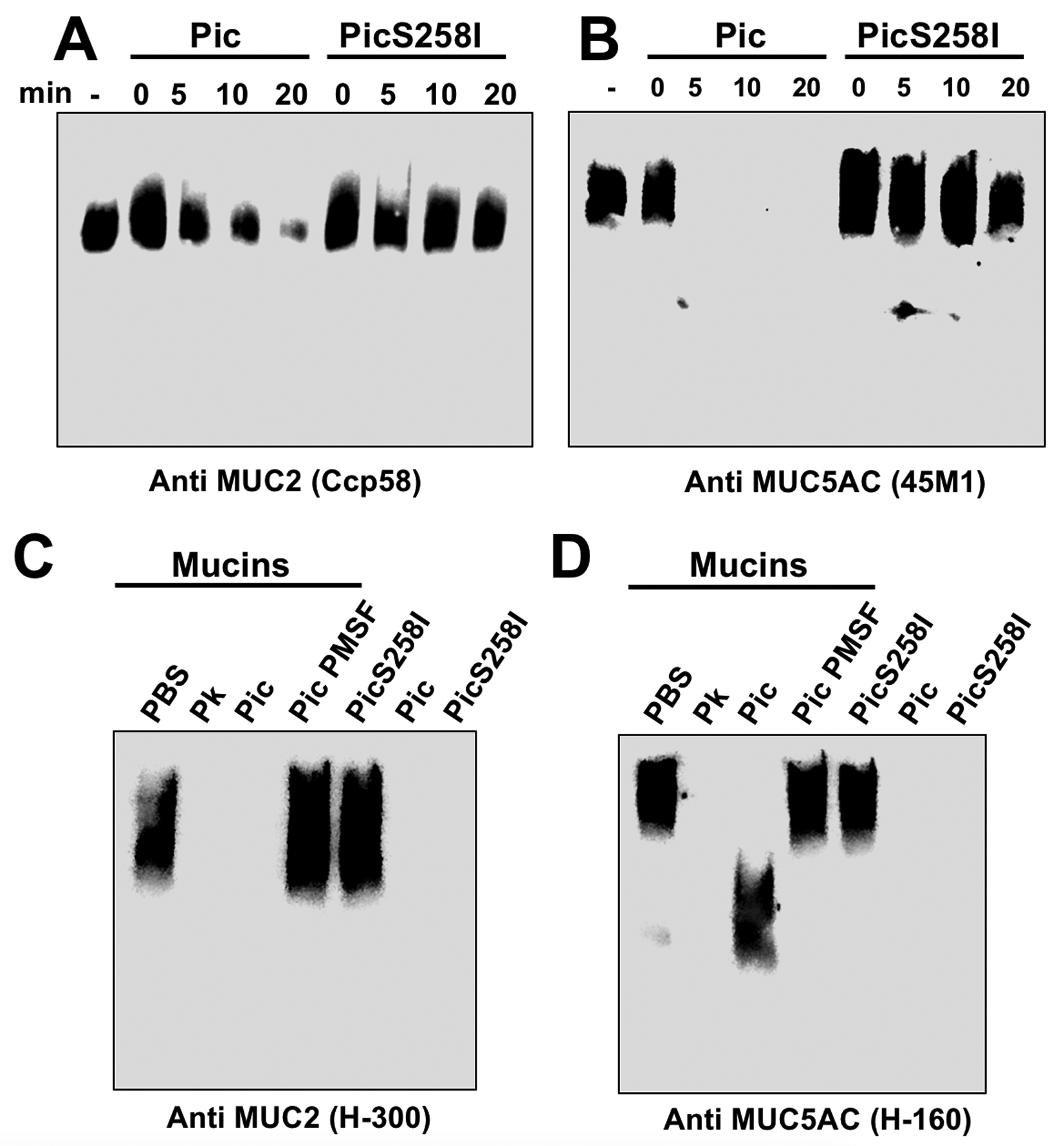

Supplement: Supplementary Figure 3 — MUC2 and MUC5AC degradation by Pic analyzed in SDS-agarose gels electrophoresis and Western blot using different antibodies. (A, B) Pic degrades more efficiently MUC5AC than MUC2. Soluble fractions from lysates of LS174T cells were incubated 2 µg/ml of Pic (A) or PicS258I (B) at different times. Mucin were electrophoresed in SDS-agarose gels and analyzed by Western blot using anti-MUC2 (Ccp58) or anti-MUC5AC (45M1) antibodies. (C, D) Detection of different MUC2 and MUC5AC epitopes using anti-MUC2 (H-300) and anti-MUC5AC (H-160) antibodies. (C) MUC2 degradation was analyzed by Western blot using an anti-MUC2 H-300 antibody to detect the C-terminal domain (1,214–1,373 aa). (D) MUC5AC degradation was analyzed by Western blot using an anti-MUC5AC H-160 antibody to detect the N-terminal domain (4,880–5,179 aa). [file Image_3.tif]

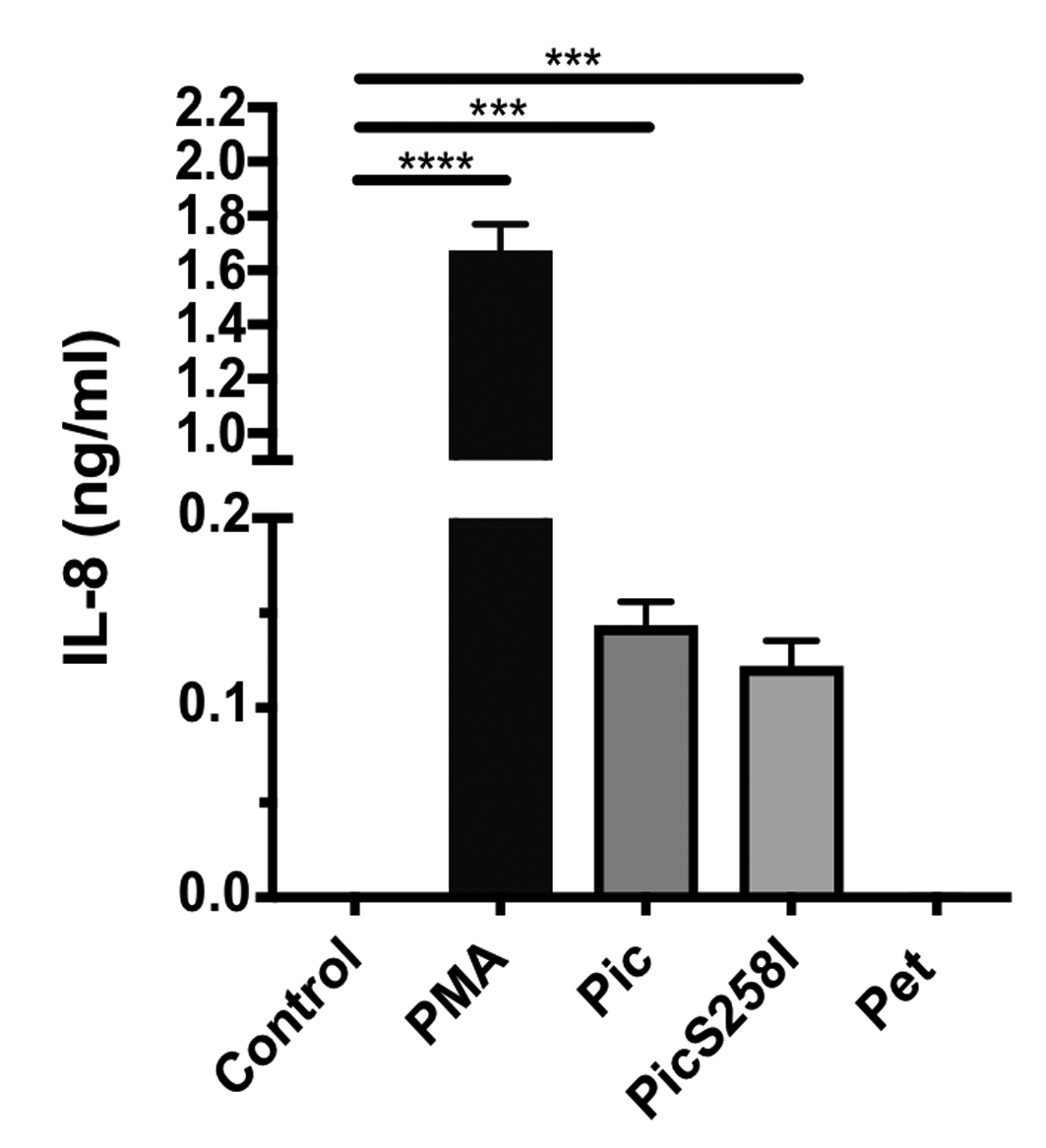

Supplement: Supplementary Figure 4 — Pic does not increase secretion of IL-1β, TNF-α, IL-6, but a slightly the IL-8 secretion. LS174T cells (500,000) were grown in 30 mm plates at 37°C for 48 h. Then, cells were kept 17 h in DMEM medium without FBS. LS174T cells were stimulated with 5 µg/ml of Pic, or PicS258I at 37°C for 4 h. After stimulations, supernatants were recovered and analyzed using a human inflammatory cytokines CBA kit (BD Biosciences). Phorbol 12-myristate 13-acetate (PMA, 2 µM) was used as a positive control and Pet protein was used as a negative control. Data were acquired in a BD FACS Fortessa flow cytometer. Statistical analysis was performed using one-way ANOVA with Dunnet’s post hoc test, ***p < 0.001%, ****p < 0.0001%. [file Image_4.tif]
